# Annexin A10 is a candidate marker associated with the progression of pancreatic precursor lesions to adenocarcinoma

**DOI:** 10.1371/journal.pone.0175039

**Published:** 2017-04-03

**Authors:** Jianhui Zhu, Jing Wu, Xiucong Pei, Zhijing Tan, Jiaqi Shi, David M. Lubman

**Affiliations:** 1 Department of Surgery, University of Michigan Medical Center, Ann Arbor, MI, United States of America; 2 Department of Toxicology, School of Public Health, Shenyang Medical College, Liaoning, China; 3 Department of Pathology, University of Michigan School of Medicine, Ann Arbor, Michigan, United States of America; Centro Nacional de Investigaciones Oncologicas, SPAIN

## Abstract

Annexins are a multigene family of calcium and phospholipid-binding proteins that play important roles in calcium signaling, cell motility, differentiation and proliferation. Our previous mass spectrometry-based proteomics study revealed that annexin A10 (ANXA10) was uniquely overexpressed in pancreatic CD24^+^ adenocarcinoma cells that were dissected from clinical PDAC tissues but was absent in CD24^-^ adjacent normal cells. The correlation between ANXA10 expression and the progression of pancreatic cancer remains unknown. In this study, we performed an immunostaining assay to evaluate ANXA10 expression in 155 primary human tissue specimens, including normal pancreas, chronic pancreatitis (CP), pancreatic adenocarcinoma (PDAC), pancreatic intraepithelial neoplasia (PanIN, the most important precursor of PDAC), and intraductal papillary mucinous neoplasm (IPMN). The immunostaining result showed that ANXA10 was significantly overexpressed in PanINs, IPMNs, and PDACs but negative in normal pancreas and the majority of chronic pancreatitis tissues. Statistical analysis revealed that ANXA10 expression was significantly associated with PDAC and its precursor lesions (p<0.0001). Abundant ANXA10 expression was predominantly present in pancreatic ductal epithelial cells of PanINs, IPMNs, and tumor cells of PDACs. Since PDAC develops through a series of PanINs which in turn arise from pancreatic ducts, the consistent overexpression of ANXA10 in ductal epithelial cells in PanINs and PDACs but negative in normal pancreatic ducts suggests that ANXA10 could serve as a potential marker indicating the presence of PDAC at its earliest precancerous stages. Double immunostaining of ANXA10 and CD24 showed that there was a large overlap between these two markers in PDAC and high-grade neoplasia lesions. The statistical analysis showed that the coexpression of ANXA10 and CD24 was significantly correlated with the progression of pancreatic precursor lesions towards PDACs.

## Introduction

Pancreatic adenocarcinoma (PDAC) is the fourth leading cause of cancer death in the United States with a 5-year survival rate of <5% [1]. Due to lack of symptoms at early stages and the rapid and aggressive course of this disease, PDAC is typically diagnosed at advanced stages and only about 15% of patients are found to be eligible for surgical resection [2]. A better outcome can be achieved if PDAC can be detected at its early stages or preferably at a precancerous stage. It is widely accepted that PDAC develops through a series of precursor lesions called pancreatic intraepithelial neoplasia (PanIN), i.e. PanIN-1, PanIN-2, and PanIN-3 [3–4]. According to a revised classification system, PanIN-1 and 2 are categorized as low-grade dysplasia and PanIN-3 as high-grade dysplasia which is also identified as carcinoma *in situ* [5]. Therefore, to identify molecular signatures specific for these precursor lesions during the progression of PDAC can promote early identification of these lesions and their changes during the progression towards cancer.

In our previous work [6], we conducted a comprehensive proteomic analysis of CD24^+^ PDAC cells procured from frozen clinical PDAC tissues, with a comparison of CD24^-^ cells isolated from patient-matched adjacent normal tissues (ANT). CD24 is a cancer stem cell (CSC) marker for PDAC [7] which plays important roles in the carcinogenesis process of the pancreas [8]. A group of annexins, including ANXA1, ANXA2, ANXA3, ANXA10, and ANAX13, were detected with significant overexpression in CD24^+^ PDAC cells compared to CD24^-^ ANT cells [6]. The annexin (ANXA) family is a multigene family of calcium and phospholipid-binding proteins [9] that possess a variety of cellular functions and play important roles in membrane transport, calcium signaling, cell differentiation and proliferation [10–12]. Extensive studies have shown that annexins consistently exhibit aberrant regulation in various types of neoplasms [13].

Changes in annexin expression or subcellular localization are highly associated with cancer development and progression [10, 13]. For example, ANXA1 as a protein related to the carcinogenesis process has been well studied and its over-expression has been observed in pancreatic adenocarcinoma where it has been correlated with poor tumor differentiation [14]. Cell surface ANXA2 expression was shown to be strongly upregulated during the progression from low-grade PanIN lesions to pancreatic adenocarcinoma [15]. Increased expression of ANXA10 was closely related to a gastric phenotype in serrated pathway to colorectal carcinoma [16] while the downregulation of ANXA10 was correlated with tumor progression in intestinal-type gastric carcinoma [17]. Compared to the other 12 members in human ANXA family, ANXA10 has distinct features including an unusual single codon deletion and loss of two main type II calcium-binding sites [9, 18], which could result in different functional consequences. Interestingly, in our previous proteomics study, ANXA10 was found uniquely overexpressed in pancreatic CD24^+^ adenocarcinoma cells but was absent in CD24^-^ adjacent normal cells [6]. The expression pattern of ANXA10 in PDAC and its precursor PanINs as well as its correlation with the progression of PDAC remains unclear.

In this study, we evaluated the immunohistochemical expression of ANXA10 in a variety of primary human tissue specimens, including normal pancreas, chronic pancreatitis, intraductal papillary mucinous neoplasm (IPMN), PDAC and PanIN tissues from different grades of progression towards PDAC. Statistical analysis was performed to investigate the correlation between ANXA10 expression versus the major clinicopathological variables, by which to determine the role of its expression in the progression of PDAC. We also conducted double immunofluorescence staining of ANXA10 and CD24 to assess the coexpression patterns of these two markers in pancreatic-related diseases as well as its relationship with disease progression. The results suggest that ANXA10 is a potential marker indicating the presence of PDAC at its earliest precancerous stages, which could not only be a useful target for early detection of PDAC but also provide novel insight into molecular features involved in the tumorigenesis of PDAC.

## Materials and methods

### Tissue specimens

All PanIN and IPMN tissue samples were obtained from the Department of Pathology of the University Hospital, Ann Arbor, MI, according to IRB approval (IRB00001996). All PanIN and IPMN specimens had an established diagnosis at the time of assessment. A total of 37 cases of PanIN and 25 cases of IPMN were used in this study, which include PanIN-1 (*n* = 20), PanIN-2 (*n* = 12), PanIN-3 (*n* = 5), low-grade IPMN (*n* = 13), and high-grade IPMN (*n* = 12). Formalin-fixed paraffin-embedded tissue samples were sliced into 5μm sections.

The tissue microarrays (TMAs) of PDAC with normal tissues as control were purchased from US Biomax Inc. (Rockville, MD), which contains 8 normal human pancreas (21–50 years, median: 35 years), 16 cancer adjacent normal pancreas (38–80 years, median: 53 years), 5 chronic pancreatitis (35–76 years, median: 61 years), and 64 cases of PDACs (31–80 years, median: 56 years) at different clinical stages (Stage I, *n* = 22; Stage II, *n* = 32; Stage III, *n* = 5; and Stage IV, *n* = 5). Tissue specimens in the TMAs originated from different donors. The clinical features of PanIN, IPMN, chronic pancreatitis, PDAC and normal pancreas specimens are summarized in **Table 1**.

**Table 1:** Clinical pathologic characteristics of pancreatic tissue specimens involved in this study (*n* = 155).

Clinicopathologic factors	*n*
**Normal Pancreas**	8
**Cancer Adjacent Normal**	16
**Chronic Pancreatitis**	5
**PanIN**	
PanIN-1	20
PanIN-2	12
PanIN-3	5
**IPMN**	
Low-grade	13
High-grade	12
**PDAC (TNM stages)**	
I	22
II	32
III	5
IV	5

### Measurement of serum ANXA10 abundance

The abundance of serum ANXA10 was measured by ELISA assay (antibodies-online, Atlanta, GA) according to the manufacturer’s instructions. Seventy nine serum samples from patients and healthy donors were provided by the University Hospital, Ann Arbor, Michigan according to IRB approval, which includes 21 chronic pancreatitis (CP), 12 intraductal papillary mucinous neoplasm (IPMN), 23 PDACs, and 15 normal controls. The pancreatic cancer group consisted of 14 early-stage and 9 late-stage PDACs. The clinical features of the patients are summarized in **S1 Table**. The ELISA standard curve was generated by a serial dilution of the standard from 1000 pg/mL to 15.6 pg/mL and a blank as 0 pg/mL. The absorbance values of the ELISA assay were read on a microplate reader (BioTek, Winooski, VT) at a wavelength of 450 nm.

### H&E staining

FFPE tissue sections were deparaffinized in xylene for 10 minutes and rehydrated in a series of alcohol solutions (100% ethanol twice, 95% ethanol, 75% ethanol). The sections were stained with hematoxylin (Sigma), a nucleus dye, rinsed with tap water, and then counterstained in eosin (Sigma) which stains the cytoplasmic material. The sections were then dehydrated with alcohol solutions, cleared in xylene and then coverslipped using a mounting media (Sigma). With H&E staining, nuclei are stained blue or purple, whereas the cytoplasm is stained pink.

### Immunostaining of ANXA10

Immunostaining was performed to evaluate the expression of ANXA10 in PanIN, IPMN, PDAC, and normal tissues, respectively. The tissue slides were deparaffinized in xylene and rehydrated through alcohol solutions. Antigen retrieval was achieved by boiling the slides in a citrate buffer at pH 6.0 (Invitrogen, Grand Island, NY) for 15 min and cooled down to room temperature prior to antibody staining.

In the immunohistochemical staining, the tissue slides were then treated with 6% H_2_O_2_, blocked with 2% BSA for 1hr, and incubated with rabbit anti-human ANXA10 antibody (1:250 dilution, Abcam, Cambridge, MA) overnight at 4°C. Then the secondary antibody, horseradish peroxidase (HRP)-conjugated anti-rabbit IgG antibody (Abcam, Cambridge, MA), was incubated with the slides for 1 hr at room temperature. The slides were washed with PBST 3 times between steps, and were then developed in 3,3’-diaminobenzidine (DAB) solution (Vector Labs, Burlingame, CA) and counterstained with hematoxylin. The immunoreactivity is shown in *brown* and the nuclei in *blue*.

In the immunofluoresence staining, the slides were treated with 2% BSA to block nonspecific binding, and then incubated with rabbit anti-human ANXA10 antibody (1:250 dilution, Abcam, Cambridge, MA) overnight at 4°C, followed by incubation with DyLight 488 conjugated anti-rabbit IgG antibody (1:200 dilution, Vector laboratories, Burlingame, CA) for 1 hr at room temperature. The isotype control was conducted in parallel to eliminate non-specific background signal from the IgG molecules of the rabbit anti-ANXA10 antibody, where a rabbit IgG isotype control (1:250 dilution, Abcam, Cambridge, MA) was applied in place of the primary ANXA10 antibody. There were 3 washes with PBST between each step. ANXA10 immunofluorescence staining is shown in *green* under the fluorescent microscope. The slides were counterstained with DAPI (Sigma) to visualize nuclei (*blue*).

### Double immunofluorescence staining of ANXA10 with CD24

Double immunofluorescence staining was performed to examine the co-expression patterns of ANXA10 with CD24 (CSC marker for PDAC) in PanIN, IPMN, PDAC, and normal tissues, respectively. The slides were deparaffinized, rehydrated, and treated with citrate buffer for antigen retrieval and then 2% BSA to block non-specific binding as described above. To achieve double IF staining of ANXA10 and CD24, rabbit anti-human ANXA10 antibody (1:250, Abcam, Cambridge, MA) was mixed with mouse anti-human CD24 antibody (1:150, Abcam, Cambridge, MA). The antibody mixture was then incubated with the slides overnight at 4°C. Subsequently, DyLight 488 anti-rabbit IgG (*green*) and DyLight 549 anti-mouse IgG (*red*) (Vector laboratories, Burlingame, CA) were diluted (1:150) and incubated with the slides for 1 hr at room temperature. Nuclei visualization was explored by DAPI counterstaining (*blue*).

### Histological assessment

The immunostaining score of ANXA10 and CD24 was calculated as described previously [19–20], based on the product of the proportion score of percentage positive cells (0 = no staining, 1 < 10%, 2 = 10–50%, 3 = 51–80%, 4 > 80%) multiplied by an intensity score (0 = negative, 1 = weak, 2 = moderate, 3 = strong).

The specimen was considered positive when the multiplication product (from 0 to 12) was ≥ 2. As for the cases of PanINs and IPMNs which were both characterized by the intraductal neoplastic proliferation or epithelial dysplasia, we assigned the scores of ANXA10 expression on pancreatic ductal cells. In PDACs, the scores were assessed for ANXA10 staining on tumor cells. The staining score for CD24 was also assessed under the same criteria. All slides were evaluated independently by two investigators including a clinical pathologist.

### Statistical analysis

The correlation between ANXA10 expression and disease stages in the progression of PDAC was studied using the analysis of covariance (ANOVA). Categorical variables in individual disease groups were compared with a χ^2^ test. The comparison of IHC scores between each group, including normal pancreas, chronic pancreatitis, low- and high-grade IPMNs/PanINs, and PDACs of early- and late-stages, was made by comparing the least square means while the multiple comparisons were adjusted by the Tukey's method. A value of *p* < 0.05 was considered statistically significant. The scatter plot of the IHC scores among groups was generated with GraphPad Prism 6 (La Jolla, CA), with reference lines of mean and SEM, which allows for an assessment of the correlation between ANXA10 expression and disease progression. The receiver operating characteristics (ROC) curve of ANXA10 expression between the disease stages was analyzed using Prism 6 (La Jolla, CA). The 2D plot of ANXA10 and CD24 staining scores in different disease groups was generated with SPSS 16.0 (IBM, Armonk, NY).

## Results

### ELISA analysis of ANXA10 in patient sera

First, we investigated the possibility of ANXA10 as a serum indicator for pancreatic diseases and evaluated ANXA10 levels in serum of patients with pancreatic ductal adenocarcinoma (PDAC), chronic pancreatitis (CP), intraductal papillary mucinous neoplasm (IPMN), and healthy donors by ELISA assay. **S1 Fig** shows the scatter plots of ANXA10 concentrations in these serum samples. The serum ANXA10 levels (mean ± SEM) in PDAC, CP, IPMN, and healthy controls were 720.7±140.4, 878.0±120.6, 865.8±171.7, and 594.0±100.2 pg/mL, respectively. The result showed that the serum level of ANXA10 was low and no significant difference in serum ANXA10 level was observed among different pancreatic disease groups, mainly because annexins lack signal sequences for secretion and are predominantly intracellular proteins [21]. The result suggests that ANXA10 is not secreted.

### Negative ANXA10 expression in normal pancreas

**Fig 1:**
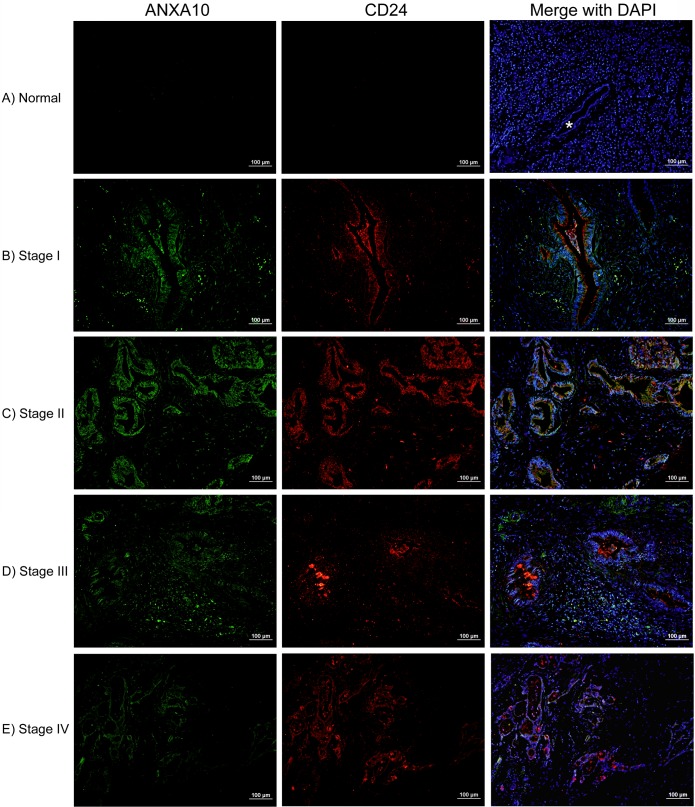
Immunofluorescence double staining with ANXA10 (*green*) and CD24 (*red*) in human normal pancreas (A) and pancreatic adenocarcinoma of early stages (B and C) and late stages (D and E). DAPI (*blue*) counterstaining was used to visualize nuclei. (A) Both ANXA10 and CD24 showed negative expression in normal pancreas. Note that normal pancreatic duct was negative for ANXA10 and CD24 (indicated by *). (B, C) Representative images of PDACs at early stages (I and II) showed strong and abundant expression of ANXA10 predominantly present on malignant pancreatic epithelial cells, exhibiting a high overlap with CD24 (*yellow* or *orange* in the merged images). (D, E) Abundant expression of ANXA10 was also observed in late-stage PDACs (stages III and IV), which was co-expressed with CD24 at some extent. A PDAC specimen at stage III (D) showed ANXA10 expression on both malignant ductal cells and stromal cells. Scale bars = 100 μm.

Immunostaining showed that there was no expression of ANXA10 in normal pancreas. No ANXA10 expression was observed in normal pancreatic acinar, ductal or islet cells. A representative image of normal pancreas with negative ANXA10 expression is shown in **Fig 1A**, where the normal pancreatic duct exhibited no expression of ANXA10 (indicated by *). In addition, CD24 also showed negative expression in normal pancreas (**Fig 1A**), which was consistent with our previous study [6].

We also examined ANXA10 expression on the histologically normal tissues adjacent to pancreatic adenocarcinoma. As shown in **S2 Fig**, ANXA10 showed rare expression on less than 0.1% cells in cancer adjacent normal tissues, which was considered as negative. In total, 15 (93.8%) of 16 adjacent normal tissues were ANXA10 negative. Double fluorescent immunostaining with CD24 showed that weak or moderate CD24 expression was observed in 7 (43.7%) of 16 adjacent normal tissues.

### ANXA10 expression in chronic pancreatitis

The immunofluorescence staining was performed to evaluate ANXA10 and CD24 expression in chronic pancreatitis (CP, n = 5). Representative images were shown in **S3 Fig** where the H&E images were provided as references. As shown in **S3 Fig**, ANXA10 showed no staining or sparse expression in chronic pancreatitis with less than 1% ANXA10+ cells, which was considered as negative as described above. Only one case of chronic pancreatitis exhibited a moderate ANXA10 expression with less than 10% ANXA10-positive cells. However, positive CD24 expression was observed in all 5 cases of chronic pancreatitis specimens investigated in this study (shown in *red* in **S3 Fig**). CD24 expression in chronic pancreatitis was heterogeneous, and about 30–50% cells were CD24-positive in these chronic pancreatitis specimens. Note that, compared to PDAC where CD24 was mainly expressed in malignant ducts [6, 20], CD24 expression in CP was mainly present in the acinar cells but absent in epithelial ducts (**S3 Fig**).

### Overexpression of ANXA10 in PDACs

Double fluorescent immunostaining was performed to evaluate ANXA10 expression in PDACs at early (I and II) and late (III and IV) stages, as well as the co-expression pattern with CD24. Tissue microarrays, which contains 64 PDACs with clinical stages (Stage I, *n* = 22; Stage II, *n* = 32; Stage III, *n* = 5; and Stage IV, *n* = 5) were used in this study. The limited number of cases of late-stage PDAC specimens is due to the fact that late-stage pancreatic cancer was already unresectable by surgery.

In PDACs, ANXA10 exhibited strong and abundant expression predominantly present on malignant epithelial cells. As shown in **Fig 1B–1E**, strong membrane and cytoplasm expression of ANXA10 (*green*) was observed in PDACs of all stages, even in early-stage PDACs. ANXA10 also showed strong expression in stromal cells in stage III PDAC specimens (**Fig 1D**). Positive ANXA10 expression was observed in 48 (75%) of 64 pancreatic adenocarcinomas. ANXA10 expression was heterogeneous in PDAC tissues, however, in most cases more than 50% of tumor cells presented ANXA10 immunoreactivity.

In addition, as shown in the merged images of **Fig 1B–1E**, the fluorescence double staining of ANXA10 (*green*) with CD24 (*red*) showed a high overlap of these two proteins (*yellow* or *orange*) in malignant ductal cells in PDACs. CD24 expression was found in 76.6% (49/64) of pancreatic adenocarcinomas. In this study, we found that tissues that were positive for ANXA10 also exhibited abundant CD24 expression in PDACs. CD24 (*red*) exhibited a strong or moderate staining on both membrane and cytoplasm of tumor cells, predominantly present on the apical membrane of malignant ducts in PDACs as reported previously [20]. Interestingly, in early-stage of PDACs, ANXA10 showed strong and dominant expression on malignant ductal cells, with a 100% overlap with CD24.

### Overexpression of ANXA10 in PanINs

**Fig 2:**
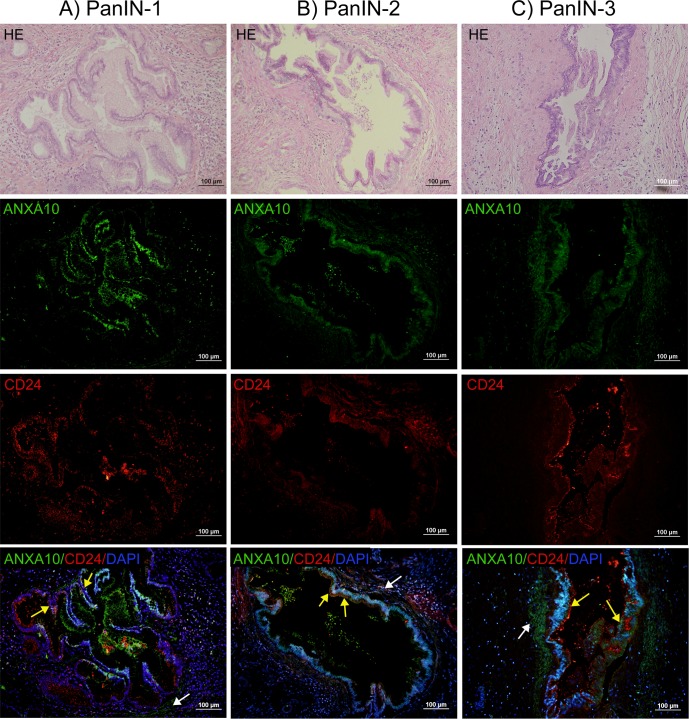
Immunofluorescence double staining showed the overlap between ANXA10 (*green*) and CD24 (*red*) in PanIN-1 (A), PanIN-2 (B), and PanIN-3 (C), respectively. The H&E staining was provided to show the morphology of PanINs. ANXA10 (*green*) showed abundant expression on the membrane and nucleus of ductal cells in PanINs. ANXA10 expression was also observed in the stromal cells, clustered around ANXA10^+^ ducts (indicated by white arrows in the merged images). CD24 (*red*) showed weak or moderate membrane/cytoplasmic staining on the epithelial ductal cells in PanINs. There was a large overlap between ANXA10 and CD24 on the ductal cells in PanINs. Scale bars = 100 μm.

We then investigated ANXA10 expression in PanINs, the most common precursor to invasive pancreatic adenocarcinoma. The result of immunohistochemical staining was shown in **S4 Fig**, where the positive immunoreactivity of ANXA10 was visualized in *brown*. ANXA10 exhibited a strong and abundant expression predominantly present on the ductal cells in PanINs. Positive ANXA10 expression in PanINs was also confirmed by immunofluoresence staining. As shown in **Fig 2**, abundant ANXA10 expression (*green*) was observed in ductal cells in PanINs, where the corresponding H&E images were provided as references. Both nuclear and membrane ANXA10 expressions were observed on the ductal epithelial cells; however, ANXA10 was absent in acinar cells. ANXA10 expression was also observed in the stromal cells in PanIN-2 and PanIN-3, clustered around ANXA10^+^ ducts (indicated by white arrows in the merged images in **Fig 2**). Positive ANXA10 expression was found in 75.7% (28/37) of PanINs, including 13 of 20 (65%) PanIN-1, 11 of 12 (91.6%) PanIN-2, and 4 of 5 (80%) PanIN-3.

Double fluorescent immunostaining of ANXA10 (green) and CD24 (red) showed that there was a large overlap between these two markers on the ductal epithelial cells in PanINs (shown in the merged images of **Fig 2**). While abundant ANXA10 expression (green) was predominantly localized on the membrane and nucleus of ductal cells in PanINs, CD24 (*red*) showed weak or moderate membrane/cytoplasmic staining on ductal epithelial cells. CD24 expression was found in 9 of 20 (45%) PanIN-1, 9 of 12 (75%) PanIN-2, and 5 of 5 (100%) PanIN-3, respectively. Statistical analysis showed that CD24 expression was significantly associated with high grade PanINs (*p* = 0.022). In addition, the co-expression between ANXA10 and CD24 was highly related with high grade PanINs.

### Overexpression of ANXA10 in IPMNs

**Fig 3:**
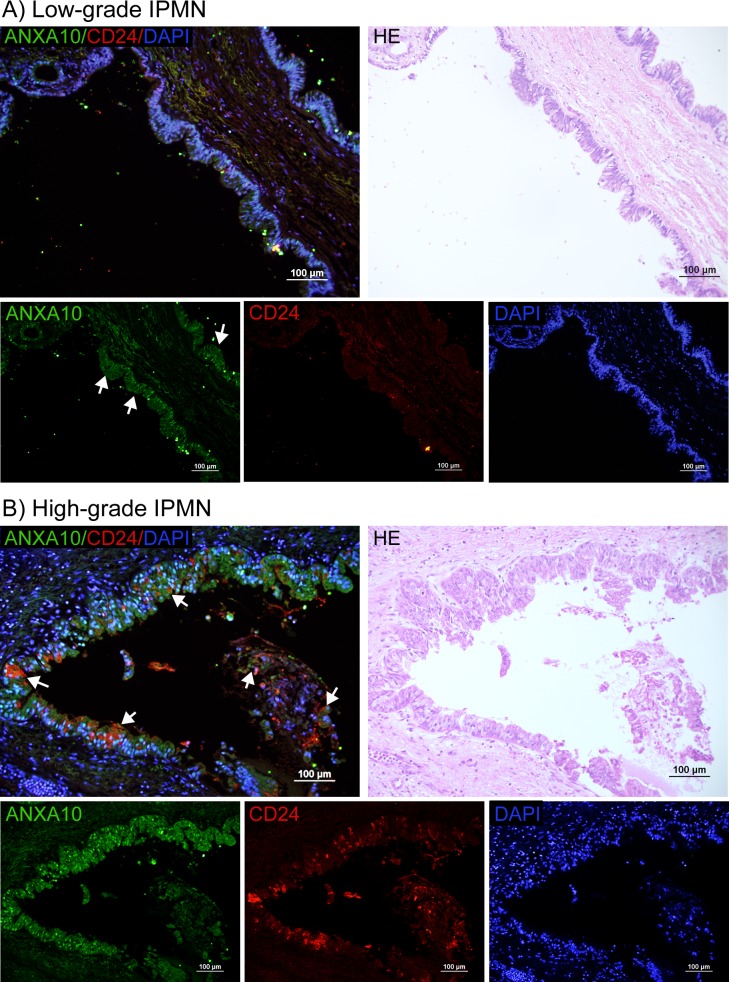
Immunofluorescence double staining of ANXA10 (*green*) and CD24 (*red*) in low- (A) and high-grade (B) IPMNs. The H&E staining was provided as a reference. (A) Strong nuclear ANXA10 (*green*) expression was observed in low-grade IPMNs, with a moderate cytoplasmic expression (indicated by arrows). (B) ANXA10 (*green*) showed abundant expression in the nuclei of ductal cells in high-grade IPMNs, presenting a large overlap with CD24 (*red*) on the epithelial ductal cells. Scale bars = 100 μm.

We also evaluated the expression of ANXA10 in 25 cases of IPMNs by fluorescent immunostaining. **Fig 3A** and **3B** show the fluorescence immunostaining of ANXA10 (*green*) and CD24 (*red*) in low- and high-grade IPMNs, respectively, as well as the merged images presenting the overlap between these two markers. H&E staining was also provided.

As shown in **Fig 3A**, in IPMNs with low-grade dysplasia, strong nuclear ANXA10 expression was observed, together with a moderate cytoplasmic expression (indicated by arrows). A minimal expression in stromal cells was also observed. In IPMNs with high-grade dysplasia, ANXA10 was predominantly present in the nucleus of IPMN cells (**Fig 3B**). ANXA10 was identified in 76.9% (10/13) of low-grade IPMNs and in 75% (9/12) of high-grade IPMNs. CD24 (*red*) showed a weak to moderate expression in low- and high-grade of IPMNs. Both cytoplasm and membrane CD24 expression was observed (**Fig 3B**). The positive rate of CD24 was low in low-grade IPMNs, which was 23.1% (3 of 13), but increased in high-grade IPMNs where positive expression of CD24 was observed in 58.3% (7 of 12). CD24 showed a moderate increasing trend according to the grade of dysplasia in IPMNs (*p* = 0.072), which was consistent with a previous study [22]. There was a large overlap between ANXA10 and CD24 in high-grade IPMNs (shown in arrows in **Fig 3B**).

We further examined the expression patterns of ANXA10 and CD24 in different histological subtypes of IPMNs. IPMN is classified into four histological subtypes (gastric, intestinal, pancreatobiliary, and oncocytic) based on its histomorphological features and mucin expression [23]. Among the 25 cases of IPMNs, there were 15 cases of gastric type (low-grade, *n* = 7; high-grade, *n* = 8), 7 cases of intestinal type (low-grade, *n* = 5; high-grade, *n* = 2), and 3 cases of pancreatobiliary type (low-grade, *n* = 1; high-grade, *n* = 2). Oncocytic-type IPMN is very rare and was not included in this study. In gastric-type IPMNs, ANXA10 was identified in all (7/7) of the low-grade IPMNs and in 75% (6/8) of the high-grade IPMNs, while CD24 expression was observed in none (0/7) of low-grade IPMNs and in 37.5% (3/8) of high-grade IPMNs where they were also positive for ANXA10. In intestinal-type IPMNs, the two cases of high-grade were positive for both ANXA10 and CD24; however, in the 5 cases of low-grade, 40% of cases were ANXA10-positive but CD24-negative and 60% of the cases were CD24-positive but ANXA10-negative. In pancreatobiliary-type IPMNs, all the 3 cases were positive for ANXA10, but CD24 positivity was only observed in the high-grade IPMNs. No significant difference in the expression of ANXA10 and CD24 was observed between subtypes of IPMNs in this sample set.

### Correlation of ANXA10 expression with clinicopathological variables

To assess the correlation between ANXA10 expression and disease types, we therefore compared the positive rate and staining score of ANXA10 between these patient groups. **Table 2** shows the positive rates of ANXA10 and CD24 in normal pancreas, cancer adjacent normal tissues (ANT), chronic pancreatitis (CP), PanINs, IPMNs, and PDACs respectively. The positive rate of ANXA10 in normal pancreas, ANT, and CP was 0%, 6.2%, 20% respectively. However, the positive rate of ANXA10 in PanINs, IPMNs and PDACs was significantly increased, which was 75.7%, 76%, and 75%, respectively.

**Table 2:** ANXA10 and CD24 positive rates as well as the co-expression rate in normal pancreas, cancer adjacent normal pancreas tissues, chronic pancreatitis, PanINs, IPMNs, and PDACs, respectively.

Pathologic factors	ANXA10 positive	CD24 positive	ANXA10/CD24 Co-expressed
**Normal Pancreas**	0% (0/8)	0% (0/8)	0% (0/8)
**Cancer Adjacent Normal**	6.2% (1/16)	43.7% (7/16)	6.2% (1/16)
**Chronic Pancreatitis**	20% (1/5)	100% (5/5)	20% (1/5)
**PanIN**			
PanIN-1	65% (13/20)	45% (9/20)	45% (9/20)
PanIN-2	91.6% (11/12)	75% (9/12)	75% (9/12)
PanIN-3	80% (4/5)	100% (5/5)	80% (4/5)
**IPMN**			
Low-grade	76.9% (10/13)	23.1% (3/13)	0% (0/13)
High-grade	75% (9/12)	58.3% (7/12)	58.3% (7/12)
**PDAC** (TNM stages)			
I	77.3% (17/22)	77.3% (17/22)	77.3% (17/22)
II	71.9% (23/32)	65.6% (21/32)	62.5% (20/32)
III	80% (4/5)	80% (4/5)	80% (4/5)
IV	80% (4/5)	80% (4/5)	80% (4/5)

**Fig 4:**
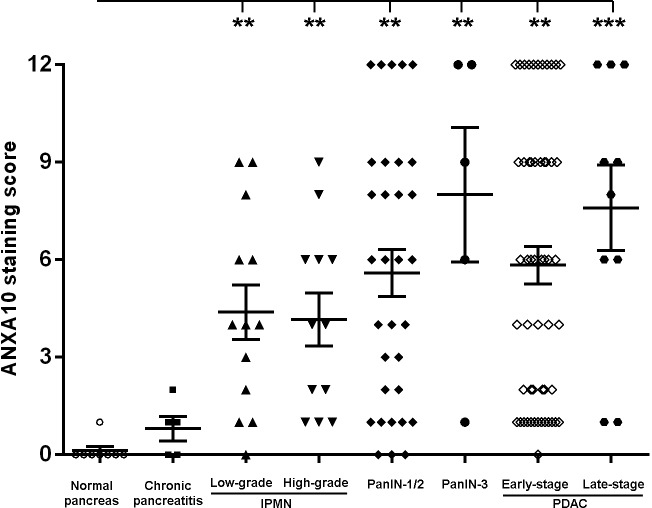
Statistical analysis of ANXA10 staining scores in normal pancreas, chronic pancreatitis (CP), low- and high-grade IPMNs, PanIN-1/2, PanIN-3, early- and late-stage PDACs, respectively. The ANXA10 staining score was distinctly higher in IPMNs, PanINs, and PDACs compared to those in normal pancreas and chronic pancreatitis cases. (**: *p*<0.01; ***: *p*<0.001)

The IHC staining score (from 0 to 12) of ANXA10 among disease groups was also evaluated. Note that ANXA10 expression was predominantly present in epithelial ducts or tumor cells in PanINs, IPMNs and PDACs, in which cases the percentage of ANXA10^+^ cells was assessed within these cells. However, ANXA10 was absent in ductal epeithial cells in normal pancreas and chronic pancreatitis, where the scores of ANXA10 were assessed in the entire tissue area. As shown in **Table 3** and **Fig 4**, the ANXA10 staining score (mean (SEM)) in normal pancreas and chronic pancreatitis was extremely low, which was 0.12 (0.12) and 0.80 (0.37), respectively. However, the staining score of ANXA10 was distinctly increased in IPMNs, PanINs, and PDACs compared to that in normal pancreas and chronic pancreatitis. The staining score (mean (SEM)) of ANXA10 in low- and high-grade IPMNs, low- and high-grade PanINs, early- and late-stage PDACs was 4.23 (0.86), 4.00 (0.83), 5.50 (0.70), 8.00 (2.07), 5.83 (0.58), and 7.60 (1.31), respectively. *P*-values were determined in ANCOVA by comparing ANXA10 staining scores between each disease group, and the multiple comparisons were performed using the Tukey's method. The overall association of disease type with ANXA10 expression yields a *p*-value less than 0.0001, indicating that ANXA10 expression is strongly associated with disease types.

**Table 3:** Correlation between ANXA10 expression and pancreatic disease types.

Characteristics	ANXA10 score (mean(SEM))	*p*-value
Disease type		<0.0001
Normal	0.12 (0.12)	
Cancer Adjacent Normal	0.44 (0.16)	
Chronic Pancreatitis	0.80 (0.37)	
IPMN	4.28 (0.58)	
PanIN	5.92 (0.69)	
PDAC	6.11 (0.53)	

The receiver operating characteristics (ROC) analysis indicated that ANXA10 expression could distinguish IPMN, PanIN, and PDAC from normal and chronic pancreatitis (AUC = 0.930, 0.931, and 0.941, respectively).

### Coexpression of ANXA10 and CD24 associated with disease progression

Since ANXA10 and CD24 were negative in normal pancreas, but a significant overlap of these two markers was observed in PanINs, high-grade IPMNs, and PDACs of all stages, we further assessed the relationship between the coexpression of ANXA10 and CD24 with disease progression. 2D plots of ANXA10 and CD24 staining scores in various disease groups, including normal pancreas, chronic pancreatitis (CP), low- and high-grade IPMNs, low- and high-grade PanINs, early- and late-stage PDACs, were generated using SPSS16.0. In total, 139 tissue specimens were involved in statistical analysis.

**Fig 5:**
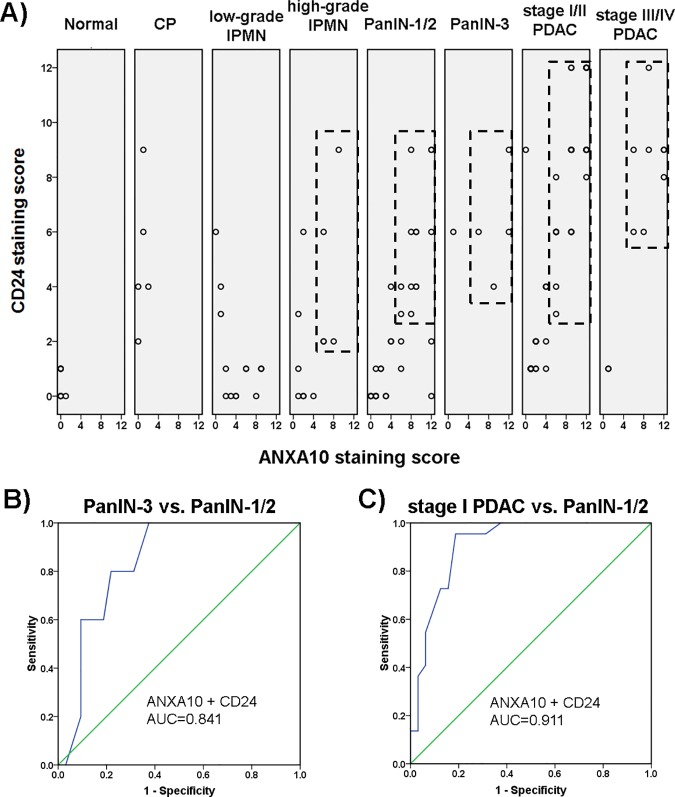
Correlation of the coexpression ANXA10 and CD24 with pancreatic diseases. (A) 2D plots of staining scores of ANXA10 (x axis) and CD24 (y axis) in different disease groups including normal pancreas, chronic pancreatitis (CP), low- and high-grade IPMNs, PanIN-1/2, PanIN-3, early- and late-stage PDACs, respectively. Each spot represents an individual tissue specimen, while spots with the same scores are overlapped. Normal pancreas tissues were found in the lower left panel of its plot, with both ANXA10 and CD24 scores less than 1 which was considered as negative expression. Chronic pancreatitis tissues were found with low ANXA10 score but higher CD24 score. However, most high-grade IPMNs, PanINs, and PDACs that exhibited distinctly increased ANXA10 expression also showed high CD24 scores, which were found in the upper right panel of their plots (highlighted in rectangles). (B and C) Receiver operating characteristics (ROC) curves of the combination of ANXA10 and CD24 scores to distinguish PanIN-3 from PanIN-1/2 (B) and to distinguish PDAC at stage I from PanIN-1/2 (C), respectively.

As shown in **Fig 5A**, Normal pancreas tissues were found in the lower left panel of the plot, with both ANXA10 and CD24 scores less than 1, which was considered as negative expression. Chronic pancreatitis tissues were found in the upper left panel of the plot, with low ANXA10 scores but high CD24 scores. However, most high-grade IPMNs, PanINs, and PDACs were clustered in the upper right panel of their plots, indicating high staining scores of both ANXA10 and CD24 (rectangles in **Fig 5A**). The result showed that high-grade IPMNs, PanINs, and PDACs that exhibited distinctly increased ANXA10 expression also presented increased CD24 expression. The statistical analysis showed that coexpression of ANXA10 and CD24 was highly correlated with PDAC and high-grade neoplasia lesions. When combining the CD24 staining score with the ANXA10 score, the performance of ANXA10 as a marker to distinguish low- and high-grade IPMNs was significantly increased (AUC = 0.702). The combination of the ANXA10 score and CD24 score also showed improved performance in differentiating PanIN-3 from PanIN-1/2 lesions (AUC = 0.841, **Fig 5B**) compared to ANXA10 alone (AUC = 0.665). Moreover, the combination of ANXA10 and CD24 scores had an AUC of 0.911 to distinguish PDACs at stage I from PanIN-1/2 lesions (**Fig 5C**).

## Discussion

Aberrant ANXA10 expression has been reported to be closely associated with adenocarcinomas of the gastric and pancreatobiliary system [17, 24] as well as colorectal carcinoma (CRC) [16, 25–26]. The ANXA10 expression was highly related to gastric phenotype in the serrated pathway to colorectal carcinoma [16] and associated with poor prognosis in CRCs [25]. With the screening of primary carcinomas of major organs, Lu et al. found that ANXA10 expression was observed in 46% of gastric, 72% of ampullary, 78% of pancreatic and 33% of biliary adenocarcinomas [24]. In addition, ANXA10 was expressed in 83% of metastatic pancreatic and 47% of metastatic gastric adenocarcinomas, but was observed in only 2% of metastatic adenocarcinomas from other organs [24]. These results indicated that ANXA10 represents a specific marker for adenocarcinomas of the upper gastrointestinal tract and pancreatobiliary origin [24]. Upregulated ANXA10 expression was also identified in our previous tissue-based proteomic study of CD24 subpopulations isolated from frozen clinical PDAC tissues [6].

The correlation of ANXA10 expression with the precursor lesions of pancreatic cancer has not yet been reported. In this study, we evaluated ANXA10 expression in pancreatic tissues during the progression of pancreatic cancer by immunostaining analysis, which included normal pancreas, cancer adjacent normal tissues, chronic pancreatitis, IPMNs (low- and high-grades), PanINs (low- and high-grades) and PDACs at early- and late-stages. ANXA10 showed no expression in normal pancreas; specifically it was absent in normal pancreatic ductal, acinar and islet cells. ANXA10 was also negative in the adjacent normal tissue to pancreatic adenocarcinoma and the majority of chronic pancreatitis tissues. However, ANXA10 was significantly overexpressed in PDACs and precancerous lesions, i.e. PanINs and IPMNs. Abundant ANXA10 expression was predominantly present in pancreatic ductal epithelial cells of PanINs and IPMNs and tumor cells of PDACs. The overall association of disease type with ANXA10 expression yields a *p*-value less than 0.0001, indicating that ANXA10 expression is strongly associated with pancreatic disease types.

A widely accepted paradigm is that PDAC develops through a series of PanINs which are classified as PanIN-1, PanIN-2, and PanIN-3 based on the degree of cellular and nuclear atypia [27]. According to the revised 2-tiered classification system for PanINs, PanIN-1 and 2 are categorized as low-grade dysplasia and PanIN-3 as high-grade dysplasia (carcinoma *in situ*) [5]. The cell of origin for PDAC is still unknown. A widely held view is that PDAC originates from pancreatic ductal cells while some studies suggest that the acinar-ductal metaplasia (ADM) might represent an origin for PDAC where the mechanism is still unclear [28]. Regardless of the specific cell origin, PDAC most likely arises from the clonal expansion of cells that acquire a growth advantage through oncogene activation [29]. In this study, ANXA10 expression was negative in normal pancreas but positive in 65% of PanIN-1, 91.6% of PanIN-2, 80% of PanIN-3, and 75% of PDACs. No ANXA10 expression was observed in normal pancreatic acinar, ductal, or islet cells; however, in PanINs, ANXA10 expression was predominantly localized on the ductal cells but absent in acinar cells. In PDACs, ANXA10 also showed a dominant expression in tumor cells within duct-like structures. ANXA10 is a member of calcium-dependent phospholipid-binding protein family which plays a role in the regulation of cellular growth and in signal transduction pathways [10]. The predominant presence of ANXA10 in pancreatic ductal cells of PanINs and tumor cells of PDACs while absent in normal pancreatic ducts suggested that ANXA10 may be involved in the early development of PanINs and promoting neoplastic progression of PanINs towards prancreatic adenocarcinoma.

Interestingly, in some cases of PanINs and PDACs, ANXA10 expression was also observed in stromal cells, clustered around ANXA10^+^ ductal cells (**Figs 1D** and **2**). PDAC is a deadly neoplasm surrounded by a dense reactive stroma called ‘desmoplastic stroma’ [30]. Numerous studies have demonstrated the importance of the interaction between tumor cells and the surrounding stroma as it plays a critical role in tumor initiation, growth and angiogenesis [30]. The positive ANXA10 expression in stroma which clustered around ANXA10^+^ ductal cells suggests that ANXA10 may be involved in the tumor-stroma interactions and promote pancreatic cancer development.

IPMN of the pancreas is an epithelial neoplasm of mucin producing cells arising in the main or branch ducts [31]. Based on cytoarchitectural atypia, IPMN is classified into low- and high-grade dysplasia [32]. IPMNs are larger than PanINs, and IPMNs tend to have more mucinous papillae than PanINs [27]. ANXA10 was identified in 76.9% of low-grade IPMNs and in 75% of high-grade IPMNs. Taken together with the fact that the pancreatic ductal epithelium in normal pancreas exhibited no expression of ANXA10, the expression of ANXA10 in IPMNs suggests that ANXA10 may be associated with IPMN development.

Interestingly, changes in subcellular localization of ANXA10 were observed between PanIN, PDAC and IPMN. In PDAC, ANXA10 expression was dominantly present on the membrane and cytoplasm of tumor cells. In PanIN, ANXA10 was predominantly localized on the membrane and nucleus of epithelial cells. However, in IPMN, ANXA10 was mainly found in the cytoplasm and nucleus of epithelial cells. Previous studies also showed that changes in annexin expression or subcellular localization are highly associated with cancer development and progression [10, 13].

CD24 is a glycosylphosphatidylinositol-anchored cell surface protein which is expressed mainly on epithelial and neural cells and strongly promotes cell adhesion, tumor growth and metastasis [33]. Various studies have reported that CD24 expression was highly correlated with the progression of pancreatic cancer [20, 22]. Our previous IHC studies showed that CD24 expression was restricted to the membrane of malignant ducts in PDACs [20] and ductal epithelial cells in PanINs [34]. In this study, abundant ANXA10 expression was also predominantly present in ductal epithelial cells of PanINs and IPMNs and malignant ducts of PDACs. Therefore, the evaluation of the coexpression of ANXA10 and CD24 could help understand the potential role of ANXA10 during the progression of pancreatic precursor lesions to adenocarcinoma.

In this study, we found that there was a high overlap between ANXA10 with CD24 in high-grade IPMNs, PanIN-3, and all stages of PDACs. The co-expression of ANXA10 and CD24 in high-grade IPMNs may be associated with higher potential of carcinogenesis. The coexpression of ANXA10 and CD24 in pancreatic ductal epithelium that occurred more frequently in high grade PanINs (i.e. PanIN-3) than in PanIN-1/2, while absent in normal pancreatic ducts, suggests that the coexistence of ANXA10 and CD24 are highly associated with the progression of PanINs. Interestingly, in early-stage of PDACs, ANXA10 showed strong and dominant expression on malignant ductal cells, with a 100% overlap with CD24. Together with the result that CD24 plays an important role in stimulating tumor cell proliferation [33], the coexpression of ANXA10 with CD24 indicates that ANXA10 may be involved in the early event during the development of pancreatic adenocarcinoma. Statistical analysis showed that the coexpression of ANXA10 and CD24 was highly correlated with the progression of pancreatic adenocarcinoma through low- to high-grade PanINs.

Note that, compared to PDAC where CD24 was mainly expressed in malignant ducts [6, 20], CD24 expression in chronic pancreatitis was not observed in epithelial ducts but in the acinar cells. Chronic pancreatitis is a progressive inflammatory disease of pancreas which is characterized by morphologic changes including fibrosis, inflammation, and loss of exocrine tissue [35]. In chronic pancreatitis, repeated inflammation causes acinar injury which eventually leads to atrophy and fibrosis [36]. CD24 as a glycosylphosphatidylinositol-anchored membrane protein also plays a role in B-cell development [33], which may help explain the immunoreactivity of CD24 in chronic pancreatitis.

## Conclusions

In summary, ANXA10 was significantly overexpressed in pancreatic ductal epithelial cells of PanINs and IPMNs and tumor cells of PDACs, but negative in normal pancreas and the majority of chronic pancreatitis. Overall, ANXA10 positive expression was observed in more than 65% of all grades/stages of PanINs, IPMNs, and PDACs including low-grade PanIN/IPMN and PDAC at stage I. The results suggest that ANXA10 expression may be a very early event in the progression of IPMNs and PanINs which can ultimately develop into invasive PDAC. ANXA10 could serve as a potential marker indicating the presence of PDAC at its earliest precancerous stages, which could be useful when powerful *in vivo* imaging/therapy techniques integrated with antibody-conjugated platforms are developed to track small changes in the pancreas. The ANXA10-positive PanINs and IPMNs were frequently positive for CD24. The co-localization of ANXA10 and CD24 in PDACs and high-grade neoplasia lesions suggests that these two markers may play roles in regulating the progression of pancreatic diseases and the development of pancreatic adenocarcinoma.

## Supporting information

S1 Fig (A) Scatter plot of serum ANXA10 concentration measured by ELISA assay in patients of CP, IPMN, PDAC, and normal controls. (B) The ELISA standard curve shows a R^2^ value of 0.9984.
(TIF)

S2 Fig Representative images showed negative ANXA10 but positive CD24 expression in cancer adjacent normal pancreas tissues. (TIF)

S3 Fig Double immunofluorescence staining of ANXA10 (*green*) and CD24 (*red*) in chronic pancreatitis (CP) with H&E images as references. DAPI counterstaining was used to visualize nuclei (*blue*). (A) A representative image of pancreatitis tissue shows rare ANXA10 but abundant CD24 expression; however both ANXA10 and CD24 were negative in pancreatic duct (indicated by arrow). (B) A pancreatitis specimen showed sparse expression of ANXA10 on the connective tissues with little overlap with CD24.
(TIF)

S4 Fig Immunohistochemical (IHC) staining of ANXA10 in PanINs. Positive immunoreactivity of ANXA10 was visualized in *brown*.
(TIF)

S1 Table Clinicopathologic characteristics of patient samples used in the ELISA assay. (DOCX)

## References

[1] Siegel RL, Miller KD, Jemal A. Cancer statistics, 2015. CA Cancer J Clin. 2015;65(1):5–29. 10.3322/caac.21254 25559415

[2] Bardeesy N, DePinho RA. Pancreatic cancer biology and genetics. Nat Rev Cancer. 2002;2(12):897–909. 10.1038/nrc949 12459728

[3] Buchholz M, Braun M, Heidenblut A, Kestler HA, Kloppel G, Schmiegel W, et al Transcriptome analysis of microdissected pancreatic intraepithelial neoplastic lesions. Oncogene. 2005;24(44):6626–36. 10.1038/sj.onc.1208804 16103885

[4] Real FX, Cibrian-Uhalte E, Martinelli P. Pancreatic cancer development and progression: remodeling the model. Gastroenterology. 2008;135(3):724–8. 10.1053/j.gastro.2008.07.033 18692502

[5] Basturk O, Hong SM, Wood LD, Adsay NV, Albores-Saavedra J, Biankin AV, et al A Revised Classification System and Recommendations From the Baltimore Consensus Meeting for Neoplastic Precursor Lesions in the Pancreas. Am J Surg Pathol. 2015;39(12):1730–41. PMCID: PMC4646710. 10.1097/PAS.0000000000000533 26559377 PMC4646710

[6] Zhu J, Nie S, Wu J, Lubman DM. Target proteomic profiling of frozen pancreatic CD24+ adenocarcinoma tissues by immuno-laser capture microdissection and nano-LC-MS/MS. J Proteome Res. 2013;12(6):2791–804. PMCID: PMC3726273. 10.1021/pr400139c 23679566 PMC3726273

[7] Li C, Heidt DG, Dalerba P, Burant CF, Zhang L, Adsay V, et al Identification of pancreatic cancer stem cells. Cancer Res. 2007;67(3):1030–7. 10.1158/0008-5472.CAN-06-2030 17283135

[8] Sagiv E, Kazanov D, Arber N. CD24 plays an important role in the carcinogenesis process of the pancreas. Biomed Pharmacother. 2006;60(6):280–4. 10.1016/j.biopha.2006.06.006 16824727

[9] Gerke V, Moss SE. Annexins: from structure to function. Physiol Rev. 2002;82(2):331–71. 10.1152/physrev.00030.2001 11917092

[10] Mussunoor S, Murray GI. The role of annexins in tumour development and progression. J Pathol. 2008;216(2):131–40. 10.1002/path.2400 18698663

[11] Rescher U, Gerke V. Annexins—unique membrane binding proteins with diverse functions. J Cell Sci. 2004;117(Pt 13):2631–9. 10.1242/jcs.01245 15169834

[12] Gerke V, Creutz CE, Moss SE. Annexins: linking Ca2+ signalling to membrane dynamics. Nat Rev Mol Cell Biol. 2005;6(6):449–61. 10.1038/nrm1661 15928709

[13] Hayes MJ, Moss SE. Annexins and disease. Biochem Biophys Res Commun. 2004;322(4):1166–70. 10.1016/j.bbrc.2004.07.124 15336964

[14] Bai XF, Ni XG, Zhao P, Liu SM, Wang HX, Guo B, et al Overexpression of annexin 1 in pancreatic cancer and its clinical significance. World J Gastroenterol. 2004;10(10):1466–70. PMCID: PMC4656286. 10.3748/wjg.v10.i10.1466 15133855 PMC4656286

[15] Esposito I, Penzel R, Chaib-Harrireche M, Barcena U, Bergmann F, Riedl S, et al Tenascin C and annexin II expression in the process of pancreatic carcinogenesis. J Pathol. 2006;208(5):673–85. 10.1002/path.1935 16450333

[16] Tsai JH, Lin YL, Cheng YC, Chen CC, Lin LI, Tseng LH, et al Aberrant expression of annexin A10 is closely related to gastric phenotype in serrated pathway to colorectal carcinoma. Mod Pathol. 2015;28(2):268–78. 10.1038/modpathol.2014.96 25081749

[17] Lu SH, Chen YL, Shun CT, Lai JN, Peng SY, Lai PL, et al Expression and prognostic significance of gastric-specific annexin A10 in diffuse- and intestinal-type gastric carcinoma. J Gastroenterol Hepatol. 2011;26(1):90–7. 10.1111/j.1440-1746.2010.06480.x 21175800

[18] Morgan RO, Jenkins NA, Gilbert DJ, Copeland NG, Balsara BR, Testa JR, et al Novel human and mouse annexin A10 are linked to the genome duplications during early chordate evolution. Genomics. 1999;60(1):40–9. 10.1006/geno.1999.5895 10458909

[19] Zhu J, Thakolwiboon S, Liu X, Zhang M, Lubman DM. Overexpression of CD90 (Thy-1) in pancreatic adenocarcinoma present in the tumor microenvironment. PLoS One. 2014;9(12):e115507 PMCID: PMC4275230. 10.1371/journal.pone.0115507 25536077 PMC4275230

[20] Jacob J, Bellach J, Grutzmann R, Alldinger I, Pilarsky C, Dietel M, et al Expression of CD24 in adenocarcinomas of the pancreas correlates with higher tumor grades. Pancreatology. 2004;4(5):454–60. 10.1159/000079824 15256807

[21] Liemann S, Lewit-Bentley A. Annexins: a novel family of calcium- and membrane-binding proteins in search of a function. Structure. 1995;3(3):233–7. 7788288 10.1016/s0969-2126(01)00152-6

[22] Ikenaga N, Ohuchida K, Mizumoto K, Yu J, Kayashima T, Hayashi A, et al Characterization of CD24 expression in intraductal papillary mucinous neoplasms and ductal carcinoma of the pancreas. Hum Pathol. 2010;41(10):1466–74. 10.1016/j.humpath.2010.04.004 20619441

[23] Furukawa T, Kloppel G, Volkan Adsay N, Albores-Saavedra J, Fukushima N, Horii A, et al Classification of types of intraductal papillary-mucinous neoplasm of the pancreas: a consensus study. Virchows Arch. 2005;447(5):794–9. 10.1007/s00428-005-0039-7 16088402

[24] Lu SH, Yuan RH, Chen YL, Hsu HC, Jeng YM. Annexin A10 is an immunohistochemical marker for adenocarcinoma of the upper gastrointestinal tract and pancreatobiliary system. Histopathology. 2013;63(5):640–8. 10.1111/his.12229 24024557

[25] Bae JM, Kim JH, Rhee YY, Cho NY, Kim TY, Kang GH. Annexin A10 expression in colorectal cancers with emphasis on the serrated neoplasia pathway. World J Gastroenterol. 2015;21(33):9749–57. PMCID: PMC4562959. 10.3748/wjg.v21.i33.9749 26361422 PMC4562959

[26] Sajanti SA, Vayrynen JP, Sirnio P, Klintrup K, Makela J, Tuomisto A, et al Annexin A10 is a marker for the serrated pathway of colorectal carcinoma. Virchows Arch. 2015;466(1):5–12. 10.1007/s00428-014-1683-6 25395067

[27] Hruban RH, Takaori K, Klimstra DS, Adsay NV, Albores-Saavedra J, Biankin AV, et al An illustrated consensus on the classification of pancreatic intraepithelial neoplasia and intraductal papillary mucinous neoplasms. Am J Surg Pathol. 2004;28(8):977–87. 15252303 10.1097/01.pas.0000126675.59108.80

[28] Reichert M, Rustgi AK. Pancreatic ductal cells in development, regeneration, and neoplasia. J Clin Invest. 2011;121(12):4572–8. PMCID: PMC3225990. 10.1172/JCI57131 22133881 PMC3225990

[29] Hezel AF, Kimmelman AC, Stanger BZ, Bardeesy N, Depinho RA. Genetics and biology of pancreatic ductal adenocarcinoma. Genes Dev. 2006;20(10):1218–49. 10.1101/gad.1415606 16702400

[30] Chu GC, Kimmelman AC, Hezel AF, DePinho RA. Stromal biology of pancreatic cancer. J Cell Biochem. 2007;101(4):887–907. 10.1002/jcb.21209 17266048

[31] Xiao SY. Intraductal papillary mucinous neoplasm of the pancreas: an update. Scientifica (Cairo). 2012;2012:893632. PMCID: PMC3820567. 24278753 10.6064/2012/893632 PMC3820567

[32] Castellano-Megias VM, Andres CI, Lopez-Alonso G, Colina-Ruizdelgado F. Pathological features and diagnosis of intraductal papillary mucinous neoplasm of the pancreas. World J Gastrointest Oncol. 2014;6(9):311–24. PMCID: PMC4163729. 10.4251/wjgo.v6.i9.311 25232456 PMC4163729

[33] Baumann P, Cremers N, Kroese F, Orend G, Chiquet-Ehrismann R, Uede T, et al CD24 expression causes the acquisition of multiple cellular properties associated with tumor growth and metastasis. Cancer Res. 2005;65(23):10783–93. 10.1158/0008-5472.CAN-05-0619 16322224

[34] Pei X, Zhu J, Yang R, Tan Z, An M, Shi J, et al CD90 and CD24 Co-Expression Is Associated with Pancreatic Intraepithelial Neoplasias. PLoS One. 2016;11(6):e0158021 PMCID: PMC4917090. 10.1371/journal.pone.0158021 27332878 PMC4917090

[35] Witt H, Apte MV, Keim V, Wilson JS. Chronic pancreatitis: challenges and advances in pathogenesis, genetics, diagnosis, and therapy. Gastroenterology. 2007;132(4):1557–73. 10.1053/j.gastro.2007.03.001 17466744

[36] Steer ML, Waxman I, Freedman S. Chronic pancreatitis. N Engl J Med. 1995;332(22):1482–90. 10.1056/NEJM199506013322206 7739686

