# Supplementary figures and images for: Annexin A10 is a candidate marker associated with the progression of pancreatic precursor lesions to adenocarcinoma
Source: PLoS One. 2017 Apr 3;12(4):e0175039. doi: 10.1371/journal.pone.0175039 (PMC5378402; doi:10.1371/journal.pone.0175039)

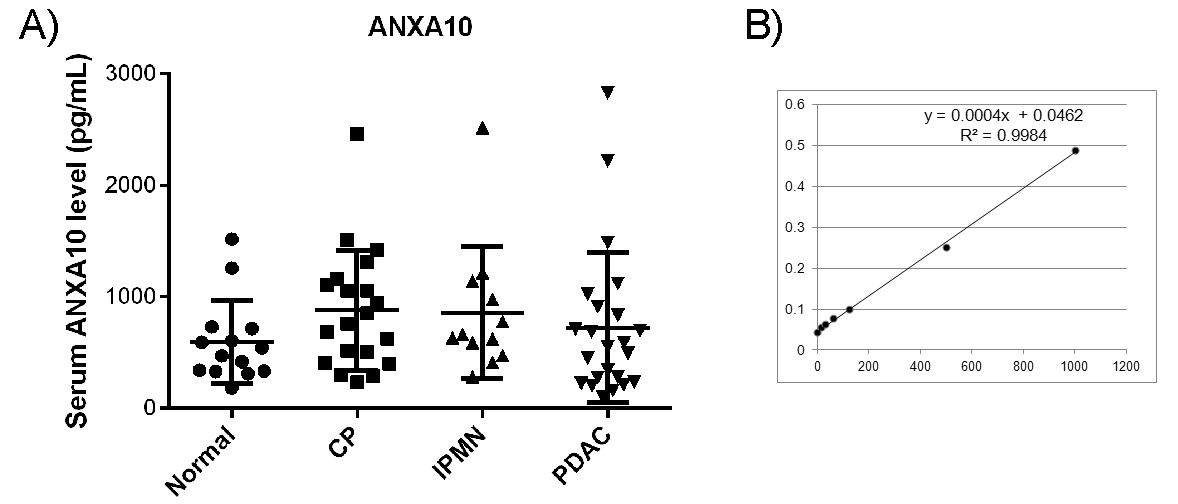

Supplement: S1 Fig — (A) Scatter plot of serum ANXA10 concentration measured by ELISA assay in patients of CP, IPMN, PDAC, and normal controls. (B) The ELISA standard curve shows a R2 value of 0.9984. (TIF) [file pone.0175039.s001.tif]

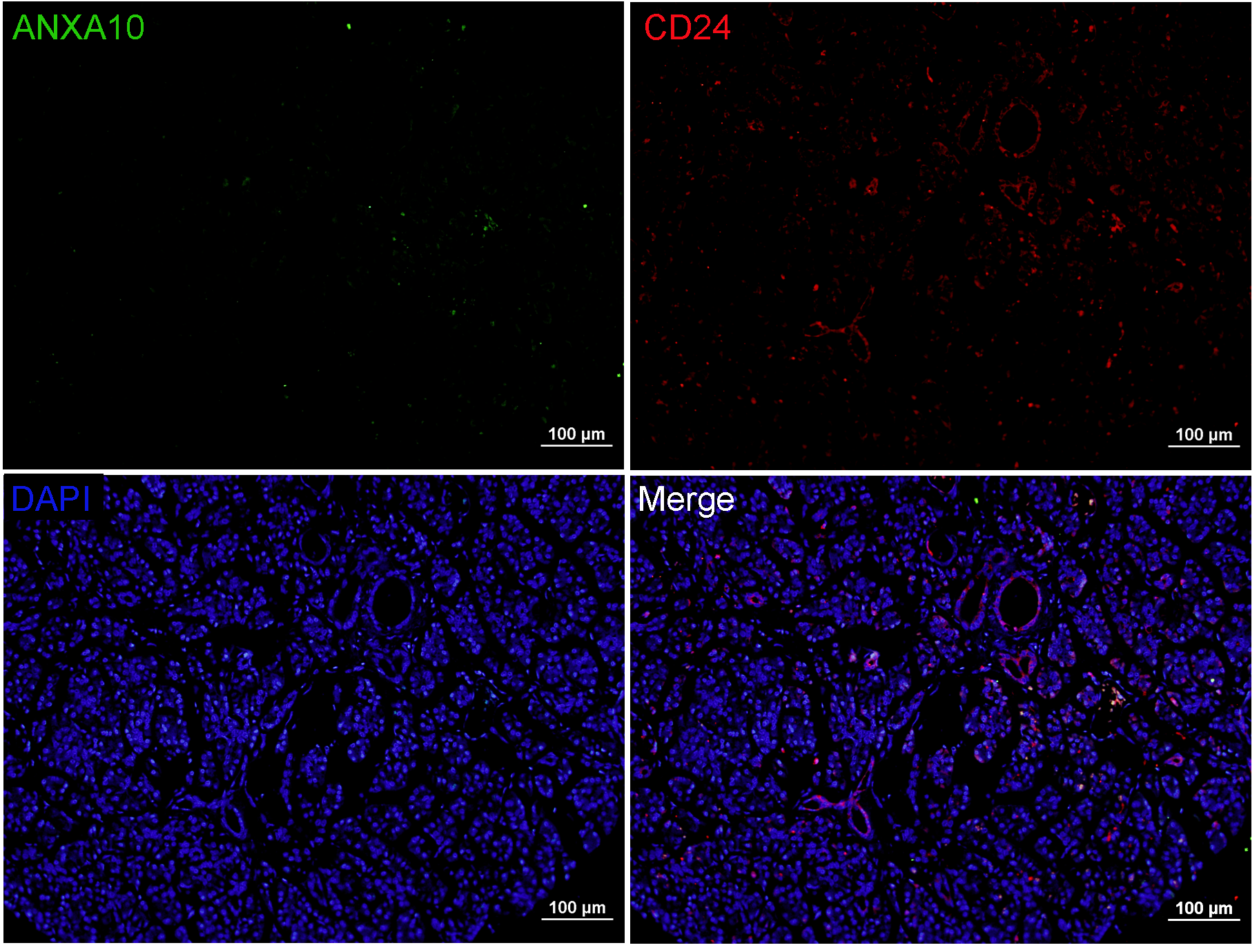

Supplement: S2 Fig — (TIF) [file pone.0175039.s002.tif]

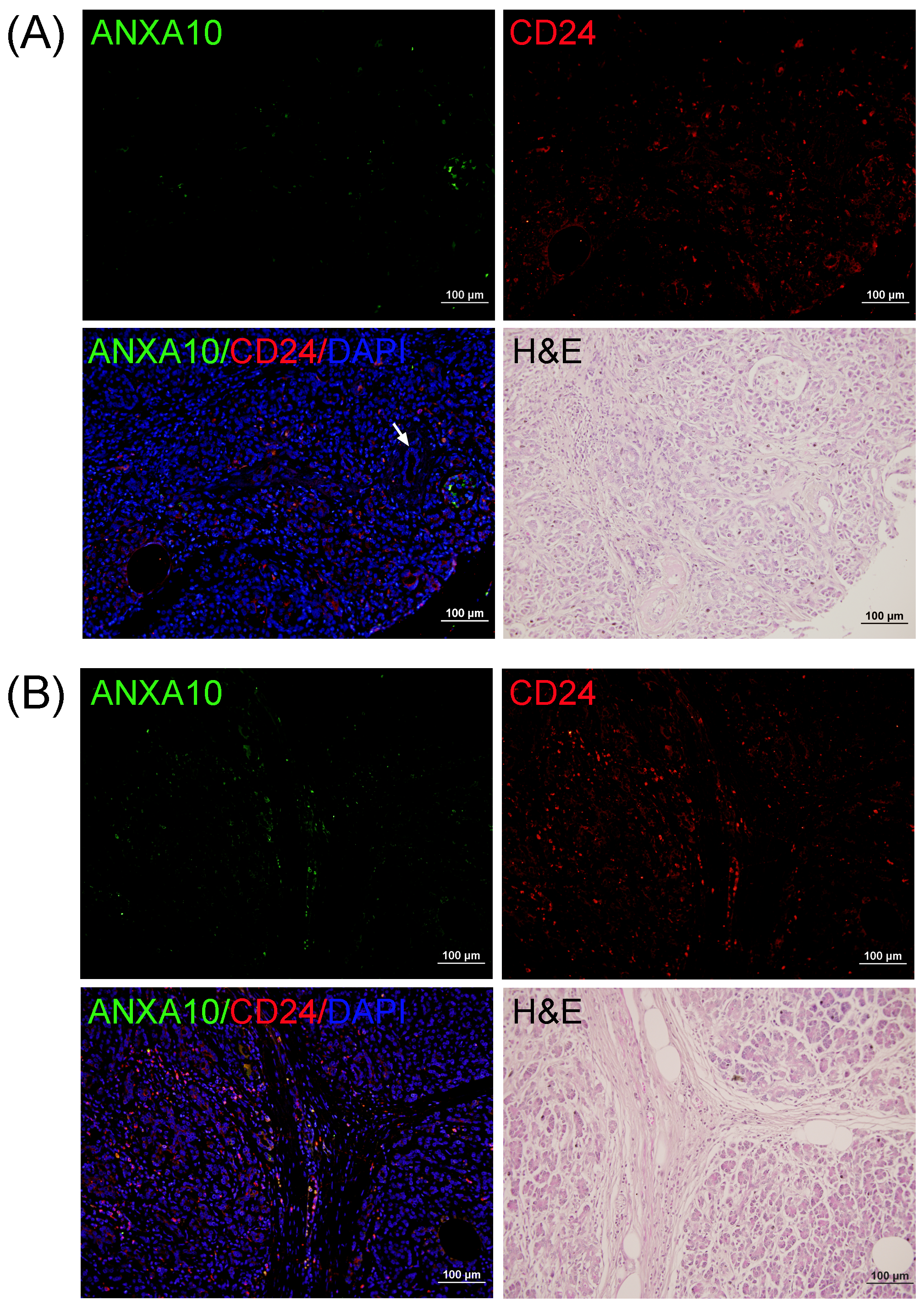

Supplement: S3 Fig — DAPI counterstaining was used to visualize nuclei (blue). (A) A representative image of pancreatitis tissue shows rare ANXA10 but abundant CD24 expression; however both ANXA10 and CD24 were negative in pancreatic duct (indicated by arrow). (B) A pancreatitis specimen showed sparse expression of ANXA10 on the connective tissues with little overlap with CD24. (TIF) [file pone.0175039.s003.tif]

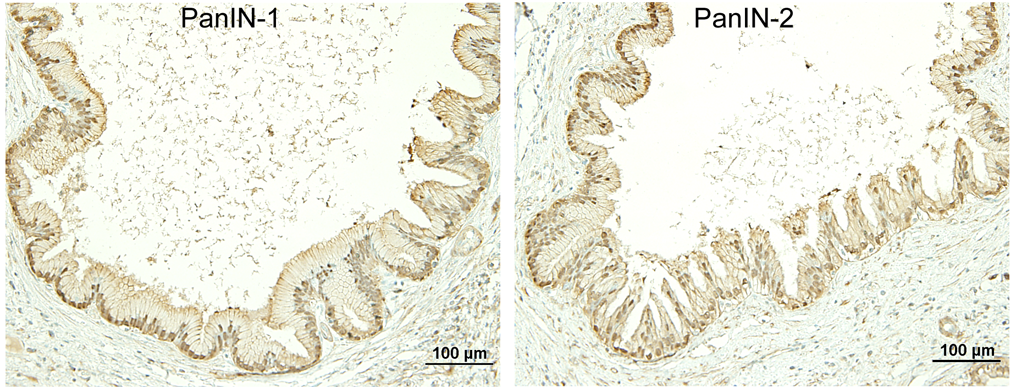

Supplement: S4 Fig — Positive immunoreactivity of ANXA10 was visualized in brown. (TIF) [file pone.0175039.s004.tif]
