# Supplementary material for: Annexin A10 is a candidate marker associated with the progression of pancreatic precursor lesions to adenocarcinoma
Source: PLoS One. 2017 Apr 3;12(4):e0175039. doi: 10.1371/journal.pone.0175039 (PMC5378402; doi:10.1371/journal.pone.0175039)
Supplement: S1 Table — (DOCX) [file pone.0175039.s005.docx]

**S1 Table.** Clinicopathologic characteristics of patient samples (*n* = 71) used in the ELISA assay.

| **Clinicopathologic factors** | ***n*** |
| --- | --- |
| **Normal** | 15 |
| **Chronic pancreatitis (CP)** | 21 |
| **Intraductal papillary mucinous neoplasm (IPMN)** | 12 |
| **Pancreatic ductal adenocarcinoma (PDAC)** |  |
| Early-stage | 14 |
| Late-stage | 9 |
